# Homocystein, Vitamin B12 and Folic Acid as Screening Biomarkers in Early Diagnosis and Gastric Cancer Monitoring

**DOI:** 10.3390/medsci12020024

**Published:** 2024-05-06

**Authors:** Fernanda Farias de Alcântara, Carla de Castro Sant’Anna, Diego Di Felipe Ávila Alcântara, Amanda de Nazaré Cohen-Paes, Paulo Cardoso Soares, Paulo Pimentel de Assumpção, Margareth Maria Braun Guimarães Imbiriba, Rommel Mario Rodriguez Burbano

**Affiliations:** 1 Ophir Loyola Hospital, Belém 66063-240, Brazil; fernanda.bmdcn@gmail.com (F.F.d.A.); santannacarla@yahoo.com.br (C.d.C.S.); diegodifelipe10@gmail.com (D.D.F.Á.A.); psoares19@hotmail.com (P.C.S.); braun.margareth@gmail.com (M.M.B.G.I.); rommel@ufpa.br (R.M.R.B.); 2 Oncology Research Center, Federal University of Pará, Belém 66073-000, Brazil; assumpcaopp@gmail.com

**Keywords:** cancer, gastric carcinogenesis, dosage, vitamin, diagnosis, screening

## Abstract

Gastric cancer has been demonstrating a reduction in the number of cases over the past decades, largely attributed to advancements in public health practices and increased accessibility to educational initiatives for the general population. Nevertheless, it persists as the third leading cause of mortality globally among both men and women. These fatalities are typically associated with delayed disease detection. The current study assessed the levels of homocysteine, vitamin B12, and folic acid as a means of establishing a screening biomarker profile that could be integrated into routine testing protocols to facilitate swift diagnosis of the illness. A total of 207 control subjects and 207 individuals with gastric cancer were scrutinized, with biochemical measurements conducted using chemiluminescence for homocysteine, folic acid, and vitamin B12. The two groups were matched based on age, tumor location, subtype, tumor classification, presence of Epstein-Barr Virus infection (EBV), and Helicobacter pylori (*H. pylori*). Significant statistical variances were identified in the mean levels of the triad of substances among cancer patients when compared to the control group for all corresponding variables. In conclusion, our study indicated that analyzing the triad of homocysteine, vitamin B12, and folic acid holds diagnostic value for gastric cancer and could potentially serve as an effective screening marker for this type of cancer in the future.

## 1. Introduction

Gastric cancer (GC) has a multifactorial etiology, meaning its incidence is influenced by several risk factors, some of which are well documented while others remain unclear in the development of the disease. Among the factors already understood, they can be divided into endogenous risk factors such as blood type A, pernicious anemia, family history of cancer, genetic variations, colon cancer, non-polyposis hereditary colorectal cancer; and exogenous factors, including diets high in sodium chloride, water and food rich in nitrous compounds, inadequate intake of vitamins C and beta carotene, smoking, alcoholism, and chronic infection by Helicobacter pylori (*H. pylori*), Epstein-Barr virus (EBV), as well as low socioeconomic status, particularly significant in regions with high incidence rates such as Latin America and Asia. Other factors remain unidentified [1,2,3,4]. The incidence of gastric cancer in the Brazilian population is high when compared to other countries, and its prevalence is even higher in the northern region of the country when compared to the other geopolitical regions of Brazil [5].

Homocysteine (He) is a small, non-essential sulfur-containing amino acid that plays a crucial role in methionine metabolism. In a recent study it was related to the imbalance of He to prostate cancer [6]. In the same year, a study was conducted that found low levels of He associated with insulin resistance [7]. Various recent studies emphasize the vital role this amino acid plays in maintaining stability and supporting various biological processes and overall health.

Vitamin B12, also known as cobalamin (Cbl), is water-soluble and not synthesized by the human body but is found in animal-based foods [8]. Individuals with gastrointestinal issues like enteritis, post-bariatric surgery, or with GC, are among those at risk for reduced absorption. Vitamin B12 deficiency can lead to hematological, neurological, and cardiovascular issues, mainly due to its impact on He metabolism and the body’s methylation reactions [9].

Folic acid or folate is crucial for synthesizing, repairing, and methylating DNA, particularly important during pregnancy and childhood to support cell division and growth. This nutrient, also known as vitamin B9, is naturally obtained from food sources [10].

Studies such as Zhang and collaborators (2015) have shown a correlation between this triad, where elevated serum He levels, folic acid, and Vitamin B12 deficiencies are linked to an increased risk of various cancers, but not for GC [11]. It’s been established that low intake of vitamin B12 and folic acid is associated with lower survival rates in GC. Given that He is recognized as an independent risk factor for diseases, it’s been found to be toxic to endothelial cells and lipoproteins due to oxygen radical generation and inflammation, creating a favorable environment for cancer [12,13].

The diagnosis of GC is challenging in many parts of the world due to the limitations of gastroscopy and the absence of reliable screening biomarkers. This leads affected patients to seek healthcare during symptomatic phases, reducing their chances of cure and survival [13,14,15]. Thus, our examination focuses on the association between patients with gastric adenocarcinoma and alterations in homocysteine, folic acid, and vitamin B12 levels, scrutinizing the behavior of these potential cancer biomarkers across all stages and in the presence of EBV and *H. pylori* infection. While similar analyses have been conducted in other studies, it is imperative to note that the Brazilian population stands out globally as one of the most genetically diverse populations. Its heterogeneous genetic composition distinguishes it significantly from other continental populations, which are typically more homogenous and extensively studied. Consequently, findings observed in these populations may not necessarily be applicable or reproducible within the context of the Brazilian population under investigation.

## 2. Patients and Methods

A total of 414 samples underwent analysis, with 207 samples from control individuals. Inclusion criteria for control subjects comprised individuals who had undergone gastric endoscopy with negative biopsy results for cancer, absence of chronic disease diagnosis, and demographic characteristics—age, gender, smoking, and alcohol consumption—matched to those of the case group. The case group comprised 207 patients diagnosed with GC and treated at Ophir Loyola Hospital, categorized according to tumor stage using the Tumor Lymph Node Metastasis (TNM) classification from the “American Joint Committee on Cancer.” TNM analyses and detection of *H. pylori* and EBV were conducted on gastric biopsies or surgical specimens from participants, who provided informed consent approved by the Research Ethics Committee of Ophir Loyola Hospital (Protocol No. 2,798,615).

Peripheral blood samples (5 mL) for vitamin B12, folate, and homocysteine measurements were collected via venipuncture in light-protected biochemistry tubes due to photosensitivity. Samples were processed at the Clinical Analysis Laboratory of the Federal University of Pará, centrifuged at 3000 RPM for 10 min to obtain serum. Serum levels of homocysteine (He), folic acid, and vitamin B12 (Cbl) were determined using chemiluminescence on a WIENER LAB CM 200 device. Chemiluminescence involves a chemical reaction generating light energy. Reactants transition to electronically excited intermediate states, releasing absorbed energy as light, termed colorimetric assays. These assays utilize antibodies linked to luminescent markers, such as luminol or acridine derivatives, employing the avidin-biotin system.

Samples were collected following sampling calculations using GraphPad Prism software version 5.0 (San Diego, CA, US). Statistical analysis utilized ANOVA for variance analysis and the Dunnet test with a 95% confidence interval (*p* ≤ 0.05) for comparing means and standard errors. A *p*-value ≤ 0.05 indicated statistical significance.

## 3. Results

The Table 1 illustrates the demographic data of the GC patients investigated. The presented characteristics include age, stomach region, histological subtype of gastric tumor for each individual, tumor classification regarding metastasis and lymph node invasion (TNM), presence or absence of EBV and/or *H. pylori* infection, and serum levels of Vitamin B12, Folic Acid, and Homocysteine.

Concerning age, individuals diagnosed with GC from the age of 28 onwards were included in the study. Statistical variations were observed between the control group and affected individuals of all ages examined for folic acid and homocysteine (*p* < 0.05). However, there was no statistically significant correlation between vitamin B12 levels in the 28 to 39-year-old range and the control group. The highest prevalence was in the age group of 60 years and above (56.04%), followed by 40–59 years with (33.33%) and 28–39 years (10.63%), respectively. Regarding tumor location, the cardia region was most prominent (34.30%), followed by the antrum region (33.33%), body (16.91%) and fundus (14.98%). With respect to subtype according to Lauren’s histology, we have intestinal cancers (52.66%) and diffuse (47.34%). Regarding the TNM classification, the highest prevalence was observed in T3N1M0 (8.7%). Most of the sample was positive for *H. pylori* (64.73%) and EBV (84.06%) infection.

Both genders displayed significant statistical differences compared to the control group. Additionally, concerning the Lauren classification, both the diffuse and intestinal subtypes exhibited statistically significant variances in levels of vitamin B12, folic acid, and homocysteine.

**Figure 1:**
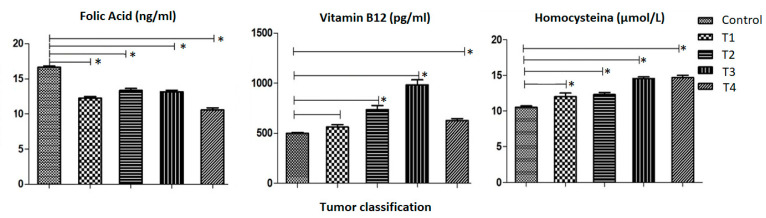
Analysis of the mean and standard error of the mean distribution of folic acid, vitamin B12 and homocysteine per ng/mL between the different tumor classes for control and GC patients. The first column of each graph represents control subjects, followed by GC patients with tumors classified as T1, T2, T3 and T4. The symbol (*) was used to represent statistically significant values (*p*-Value ≤ 0.05). The x-axis of each graph shows tumor classification and the y-axis shows serum levels of Folic Acid, Vitamin B12 and Homocystein, respectively.

Figure 1 shows a comparison of the serum levels of each of the chemical biomarkers (homocysteine, vitamin B12 and folic acid), in relation to the tumor classification found in GC patients and control subjects. In the first graph, mean values for folic acid across various cancer stages are depicted, with the control mean value recorded at 16.65 (±0.17) ng/mL. Among the case group, the average folic acid levels for patients categorized as T1 were 12.25 (±0.21) ng/mL, T2 were 13.36 (±0.25) ng/mL, T3 were 13.18 (± 0.20) ng/mL, and T4 were 10.59 (±0.29) ng/mL. Concerning vitamin B12, the control group exhibited a mean value of 499.2 (±9.43) pg/mL; whereas, patients classified as T1 had a mean value of 562.4 (±23.04) pg/mL, T2 had 736.2 (±41.51) pg/mL, T3 had 984.4 (±49.31) pg/mL, and T4 had 625.6 (±21.31) pg/mL. Graph 3 illustrates mean values for homocysteine, indicating a mean value in the control group of 10.53 (±0.20) µmol/L, while patients with T1 tumors exhibited 12.01 (±0.49) µmol/L, T2 tumors exhibited 12.31 (±0.27) µmol/L, T3 tumors exhibited 14.57 (±0.20) µmol/L, and T4 tumors exhibited 14.70 (±0.30) µmol/L.

**Figure 2:**
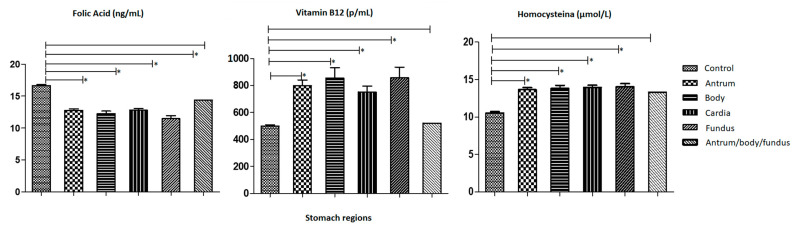
Analysis of the mean and standard error of the mean of the distribution of Folic Acid, Vitamin B12 and Homocysteine between the regions of the stomach using the one-criterion ANOVA test, and then the Dunnett test for comparison between the columns with a 95% confidence interval (*p*-Value ≤ 0.05). The first column is made up of control patients collated with the following columns: the second column is made up of the Antrum region; the third column is made up of the Body region; the fourth column is made up of the Cardia region; the fifth column is made up of the Fundus region and the sixth column is made up of the Antrum/Body/Fundus region. The symbol (*) was used to represent statistically significant values (*p*-Value ≤ 0.05). The X-axis of each graph shows the regions of the stomach tumor and the Y-axis shows the serum levels of Folic Acid, Vitamin B12 and Homocystein, respectively.

Figure 2 illustrates a comparison of the serum levels of each of the chemical biomarkers (homocysteine, vitamin B12 and folic acid), in relation to the stomach regions in GC patients and control subjects.

For folic acid, represented in the first graph, there are the average values in affected individuals according to tumor region, for the Antrum 12.78 (±0.22) ng/mL, for the Body region 12.26 (±0.39) ng/mL, for the Cardia region 12.80 (±0.25) ng/mL and for the Fundus region 11.47 (±0.46) ng/mL. For the control group, the average folic acid level was 16.65 (±0.17) ng/mL. In terms of Vitamin B12 levels, the mean value for control patients was 499.2 (±9.43) pg/mL, the value for GC patients with an Antrum tumor 799.5 (±41.54) pg/mL; the Body region with a value of 856.3 (±76.38) pg/mL; the Cardia region with a value of 753.3 (±42.64) pg/mL; the Fundus region with a value of 857.6 (±79.62) pg/mL and the Antrum/Body/Fundus region with a value of 520.9 (±0.00) pg/mL. Finally, homocysteine levels per region of the stomach tumor were 10.53 (±0.20) µmol/L in the control group; 13.66 (±0.25) µmol/L in the Antrum, 13.80 (±0.41) µmol/L in the Body, 13.95 (±0.28) µmol/L in the Cardia; 14.02 (±0.43) µmol/L in the Fundus region and 13.32 µmol/L (±0.00) in the antrum/body/fundus. Subjects affected by the tumor region were assessed for vitamin B12, folic acid, and homocysteine, revealing a statistical difference in the control group compared to the regions of the antrum, body, cardia, and fundus. However, no statistical distinction was found between the control group and the antrum/body/fundus regions.

**Figure 3:**
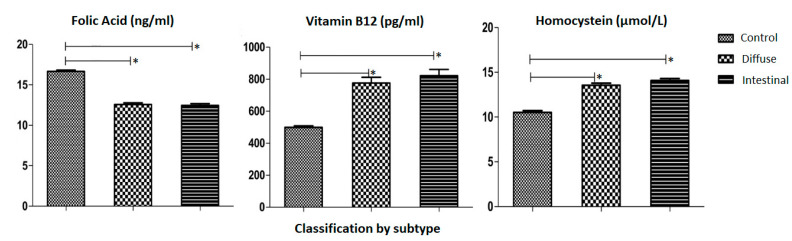
Analysis of the mean and standard error of the mean of the distribution of vitamin B12 per pg/mL, folic acid per ng/mL and homocysteine per µmol/L by tumor subtype using the one-criterion ANOVA test, followed by the Dunnett test for comparison between columns with a 95% confidence interval (*p* ≤ 0.05). The first column of each follow-up is made up of individuals from the control group, followed by the columns representing GC patients with the Diffuse subtype and the Intestinal subtype. The symbol (*) was used to represent statistically significant values.

In regard to the serum levels of markers and histological subtype, our data demonstrated that the mean levels of Folic Acid in control individuals were 16.65 (±0.17) ng/mL, in GC patients with the diffuse subtype it was 12.57 (±0.20) ng/mL, and in the Intestinal subtype it was 12.45 (±0.22) ng/mL (Figure 3). For Vitamin B12, control individuals exhibited a mean of 499.2 (±9.43) pg/mL, 776.3 (±36.03) pg/mL for the Diffuse subtype group, and 822.4 (±39.07) pg/mL for the Intestinal subtype. Regarding Homocysteine levels, the control group registered 10.53 (±0.20) µmol/L, 13.56 (±0.23) µmol/L for patients with the Diffuse subtype, and 14.09 (±0.21) µmol/mL for the Intestinal subtype.

**Figure 4:**
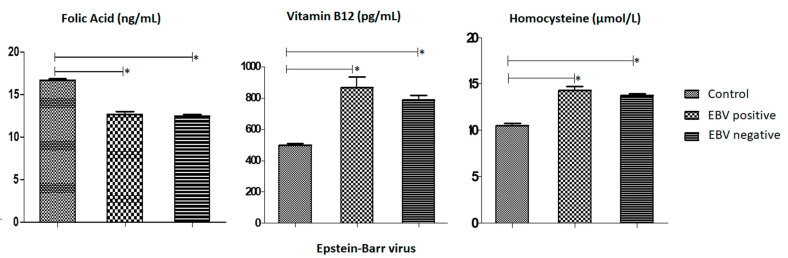
Analysis of the mean and standard error of the mean of the distribution of vitamin B12 per pg/mL, folic acid per ng/mL and homocysteine per µmol/L in individuals affected and Positive for the EBV virus and individuals affected and Negative for the EBV virus was found using the one-criterion ANOVA test, followed by the Dunnett test to compare the columns with a 95% confidence interval (*p* ≤ 0.05). The first column of each follow-up is made up of control patients collated with the following columns, the second column being made up of affected individuals and Positive for the EBV virus and the third column of each follow-up being made up of affected individuals and Negative for the EBV virus. The symbol (*) was used to represent statistically significant values (*p*-Value ≤ 0.05). The X-axis of each graph shows the EBV serology and the Y-axis the serum levels of Folic Acid, Vitamin B12 and Homocystein, respectively.

Regarding EBV serology, we compared individuals affected with the virus in relation to the distribution of the levels of each biochemical marker (Figure 4). The mean level of Folic Acid in individuals with GC and positive for EBV was 12.68 (SD ± 0.32) ng/mL. The mean level of Vitamin B12 in individuals with GC and positive for was 866.0 (SD ± 67.83) pg/mL. Finally, the mean level of Homocysteine in individuals with GC and positive for EBV was 14.32 (±0.40) µmol/L.

**Figure 5:**
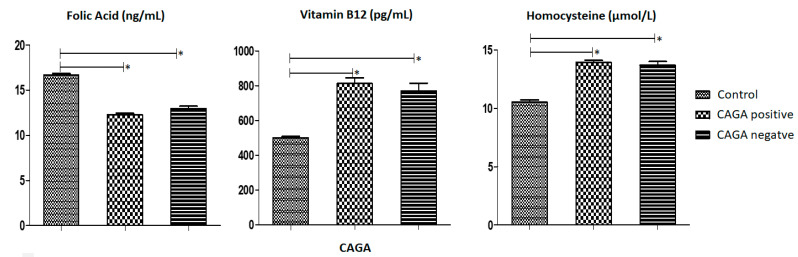
Analysis of the mean and standard error of the mean of the distribution of vitamin B12 per pg/mL, folic acid per ng/mL and homocysteine per µmol/L in affected and CAGA-positive individuals and affected and CAGA-negative individuals using the one-criterion ANOVA test, followed by the Dunnett test to compare the columns with a 95% confidence interval (*p* ≤ 0.05). The first column of each follow-up is made up of control patients collated with the following columns, the second column being made up of affected and CAGA-positive individuals and the third column of each follow-up being made up of affected and CAGA-negative individuals. The symbol (*) was used to represent statistically significant values. The X-axis of each graph shows the CACG levels, representing positive or negative for *H. pylori* infection, and the Y-axis the serum levels of Folic Acid, Vitamin B12 and Homocystein, respectively.

Finally, we conducted an analysis of the levels of Folic Acid, Vitamin B12, and Homocysteine in relation to the presence or absence of *H. pylori* infection as measured by CACG (Figure 5). The mean level of folic acid in individuals with GC and positive for *H. pylori* was 12.26 (SD ± 0.18) ng/mL. The mean level of Vitamin B12 in individuals with GC and positive for *H. pylori* was 812.0 (±33.45) pg/mL. The mean level of Homocysteine in individuals with GC and positive for *H. pylori* was 13.93 (±0.18) µmol/L. Patients with GC, both with and without EBV infection, demonstrated statistically significant differences in vitamin B12, folic acid, and homocysteine levels compared to the control group. This disparity was also observed in individuals with CAGA-positive (*H. Pylori*) GC and negatives, revealing statistical variances in vitamin B12, folic acid, and homocysteine levels when compared to the control group.

## 4. Discussion

The analysis of the triad rates has always been altered in relation to the control group. These findings corroborate Yuan-Yuan 2016 [6], which emphasized the increase in He related prostate cancer. In a recent study, plasma elevation of homocysteine in low-risk rectal adenoma, high-risk rectal adenoma, and stage I-IV rectal cancer [16]. Regarding the control group, studies support our findings by linking elevated levels of He and folic acid deficiency to an increased risk of gastric cancer [11]. A meta-analysis carried out in 2018 including 13 studies with patients diagnosed with cancers of the gastrointestinal tract demonstrated that each increase of 5 μmol/L in He levels increases the risk of these neoplastic types by approximately 7% [17]. Therefore, we infer that He concentration may be a potential biomarker for the occurrence of GC.

Miranti in 2017 [18] also identified an increased risk of developing cancer up to 5.8 times more than the control when patients had a low vitamin B12 concentration, presuming that the low intake of this vitamin would be due to an initial gastritis, for example, since, for satisfactory absorption, an intact gastric mucosa is required to produce acid and intrinsic factor. Corroborating our finding, Loedin and Speijer highlight that B12 deficiency enhances the incorrect incorporation of uracil into the genetic code, leading to impaired DNA synthesis and increasing genomic instability [19]. Furthermore, it is known that low levels of Vitamin B12 are also associated with global DNA hypomethylation, a known hallmark of early carcinogenesis [20].

Concerning the mean values of folic acid in control group patients were consistently lower compared to the control group in our study. Similar results were obtained by Lee in 2014 [21], where serum folic acid levels were significantly associated with the development of gastric cancer, as well as invasive phenotypes of this type of cancer, such as serous, lymphatic, invasion, and hepatic metastasis. Another study conducted under similar conditions tested the levels of the three markers. Their results align with those found in our analyses, indicating that the median plasma folic acid values were substantially lower than those of the controls, alongside higher homocysteine and vitamin B12 values. This profile showed a significant association with upper gastrointestinal cancer [22]. A review carried out in 2022 investigated folic acid levels and the risk of colorectal cancer in studies published from 1994 to 2022 and demonstrated that folic acid is a modulator of the risk of developing colorectal cancer [23].

Our data also demonstrated that individuals with GC, both with and without EBV infection, demonstrated statistically significant differences in vitamin B12, folic acid, and homocysteine levels compared to the control group. To date, limited research has focused on EBV + *H. pylori* co-infection [24], and no study has comprehensively assessed this co-infection alongside the levels of Folic Acid, Vitamin B12, and Homocysteine in gastric cancer. It is established that *H. pylori* infection correlates with impaired absorption of Vitamin B12 and Folic Acid, resulting in elevated Homocysteine levels due to disruption of the remethylation pathway [25,26,27]. Similarly, EBV infection is recognized for its association with gastric cancer development [28]. Dávila-Collado and colleagues (2020) examined EBV and *H. Pylori* co-infection in non-malignant gastroduodenal conditions, underscoring the importance of such investigations for gastrointestinal tract disorders at large [24]. In essence, clinical and epidemiological evidence, though from separate analyses, suggests that EBV and *H. pylori* infections correlate with various gastrointestinal disorders, emphasizing the potential significance of combined research efforts.

Our findings are noteworthy and corroborate existing literature, indicating the involvement of homocysteine, vitamin B12, and folic acid in gastric carcinogenesis modulation, with potential implications as biomarkers. Furthermore, we elucidate that viral and bacterial infections, like EBV and *H. pylori* co-infection, may contribute to serum biochemical marker imbalances, which could hold clinical screening and diagnostic relevance in gastric cancer.

## 5. Conclusions

The early diagnosis of gastric cancer poses a significant challenge in current medical practice. Within this context, our research has revealed notable variations in the levels of homocysteine, vitamin B12, and folic acid across various demographic and clinical parameters, including sex, cancer subtype, tumor stage, and the presence of EBV and/or *H. pylori* infection. Notably, these differences were observed across a wide range of ages and tumor locations. This underscores the potential utility of assessing this triad of biomarkers as part of routine clinical screening protocols for the prompt detection of GC.

## Figures and Tables

**Table 1:** Demographic characteristics of the patients diagnosed with GC investigated in the study. The results are subdivided by male, female, but also aggregated for the total number of individuals exhibiting a particular characteristic.

CATACTERISTICS	MEN (%)	WOMEN (%)	TOTAL (%)
**AGE**			
28–39	12 (9.23%)	10 (12.99%)	22 (10.63%)
40–59	40 (30.77%)	29 (37.66%)	69 (33.33%)
≥60	78 (60%)	38 (49.35%)	116 (56.04%)
**STOMACH REGION**			
Antrum	46 (35.38%)	23 (29.87%)	69 (33.33%)
Antrum/Body/Fundus	1 (0.77%)	0 (0%)	1 (0.48%)
Body	23 (17.69%)	12 (15.58%)	35 (16.91%)
Cardia	43 (33.08%)	28 (36.36%)	71 (34.30%)
Fundus	17 (13.08%)	14 (18.18%)	31 (14.98%)
**HISTOLOGICAL SUBTYPE**			
Diffuse	66 (50.77%)	32 (41.56%)	98 (47.34%)
Intestinal	64 (49.23%)	45 (58.44%)	109 (52.66%)
**TUMOR CLASSIFICATION**			
T1N0M0	3 (2.31%)	2 (2.6%)	5 (2.42%)
T1N1M0	6 (4.62%)	2 (2.6%)	8 (3.86%)
T1N1M1	2 (1.54%)	5 (5.19%)	7 (3.38%)
T2N0M0	4 (3.08%)	4 (4.19%)	8 (3.86%)
T2N1M0	7 (5.38%)	3 (3.9%)	10 (4.83%)
T2N1M1	6 (4.62%)	2 (2.6%)	8 (3.86%)
T2N2M0	2 (1.54%)	3 (3.9%)	5 (2.42%)
T2N2M1	4 (3.08%)	4 (5.19%)	8 (3.86%)
T2N3M0	4 (3.08%)	1 (1.3%)	5 (2.42%)
T2N3M1	2 (1.54%)	1 (1.3%)	3 (1.45%)
T3N0M0	10 (7.69%)	0 (0%)	10 (4.83%)
T3N1M0	9 (6.92%)	9 (11.69%)	18 (8.70)
T3N1M1	9 (6.62%)	8 (10.39%)	17 (8.21%)
T3N2M0	6 (4.62%)	3 (3.9%)	9 (4.35%)
T3N2M1	11 (8.46%)	6 (7.79%)	17 (8.21%)
T3N3M0	6 (4.62%)	1 (1.3%)	7 (3.38%)
T3N3M1	9 (6.92%)	3 (3.9%)	12 (5.8%)
T4N1M0	3 (2.3%)	3 (3.9%)	6 (2.9%)
T4N1M1	10 (7.69%)	7 (9.09%)	17 (8.21%)
T4N2M0	8 (6.15%)	2 (2.6%)	10 (4.83%)
T4N2M1	3 (2.31%)	4 (5.19%)	7 (3.38%)
T4N3M0	2 (1.54%)	3 (3.9%)	5 (2.42%)
T4N3M1	4 (3.08$)	1 (1.3%)	5 (2.42%)
**EBV SEROLOGY**			
Positive	22 (16.92%)	11 (14.29%)	33 (15.94%)
Negative	108 (83.08%)	66 (85.71%)	174 (84.06%)
**CAGA (*H. pylori* INFECTION)**			
Positive	90 (69.23%)	44 (57.14%)	134 (64.73%)
Negative	40 (30.76%)	33 (42.85%)	73 (35.26%)
**BIOCHEMICAL MARKER**			
Vitamin B12 Mean (Standard deviation)	794.94 (SD = 378.29)	810.20 (SD = 396.49)	800.61 (SD = 384.27)
Folic Acid Mean (Standard deviation)	12.55 (SD = 2.29)	12.44 (SD = 2.04)	12.51 (SD = 2.2)
Homocystein Mean (Standard deviation)	13.71 (SD = 2.37)	14.05 (SD = 2.18)	13.84 (SD = 2.3)

## Data Availability

Data are contained within the article.
